# Biofabrication of Gingival Fibroblast Cell-Laden Collagen/Strontium-Doped Calcium Silicate 3D-Printed Bi-Layered Scaffold for Osteoporotic Periodontal Regeneration

**DOI:** 10.3390/biomedicines9040431

**Published:** 2021-04-16

**Authors:** Chen-Ying Wang, Yung-Cheng Chiu, Alvin Kai-Xing Lee, Yun-An Lin, Ping-Yi Lin, Ming-You Shie

**Affiliations:** 1 Graduate Institute of Clinical Dentistry, School of Dentistry, National Taiwan University, Taipei 106319, Taiwan; jybean@gmail.com (C.-Y.W.); ann07130106@gmail.com (Y.-A.L.); 2 Division of Periodontics, Department of Dentistry, National Taiwan University Hospital, Taipei 106319, Taiwan; 3 School of Medicine, China Medical University, Taichung City 40447, Taiwan; ycchiu@me.com (Y.-C.C.); Leekaixingalvin@gmail.com (A.K.-X.L.); 4 Department of Orthopedic Surgery, China Medical University Hospital, Taichung City 40447, Taiwan; 5 x-Dimension Center for Medical Research and Translation, China Medical University Hospital, Taichung 40447, Taiwan; 6 Department of Dentistry, Far Eastern Memorial Hospital, New Taipei City 220, Taiwan; rhyne.lin@gmail.com; 7 School of Dentistry, China Medical University, Taichung City 40447, Taiwan; 8 Department of Bioinformatics and Medical Engineering, Asia University, Taichung City 41354, Taiwan

**Keywords:** collagen, strontium, gingival fibroblast, bioprinting, osteoporotic

## Abstract

Periodontal disease is a chronic disease that can lead to lose teeth and even tooth loss if left untreated. Osteoporosis and periodontal disease share similar characteristics and associated factors. Current regenerative techniques for periodontal diseases are ineffective in restoring complete function and structural integrity of periodontium due to unwanted migration of cells. In this study, we applied the concept of guided tissue regeneration (GTR) and 3D fabricated gingival fibroblast cell-laden collagen/strontium-doped calcium silicate (SrCS) bi-layer scaffold for periodontal regeneration. The results revealed that the bioactive SrCS had a hydroxyapatite formation on its surface after 14 days of immersion and that SrCS could release Sr and Si ions even after 6 months of immersion. In addition, in vitro results showed that the bi-layer scaffold enhanced secretion of FGF-2, BMP-2, and VEGF from human gingival fibroblasts and increased secretion of osteogenic-related proteins ALP, BSP, and OC from WJMSCs. In vivo studies using animal osteoporotic models showed that the 3D-printed cell-laden collagen/SrCS bi-layer scaffold was able to enhance osteoporotic bone regeneration, as seen from the increased Tb.Th and BV/TV ratio and the histological stains. In conclusion, it can be seen that the bi-layer scaffolds enhanced osteogenesis and further showed that guided periodontal regeneration could be achieved using collagen/SrCS scaffolds, thus making it a potential candidate for future clinical applications.

## 1. Introduction

Periodontal regeneration used in the surgical treatment of periodontitis is one of the earliest tissue engineering methods applied in medical specialties. Periodontitis is a microbial-associated, host-mediated inflammation. The inflammation may lead to loss of periodontal attachment and even loss of the tooth [1]. In addition, the inflammation not only destroy periodontal tissues, but it also increases the risk of future systemic diseases such as cardiovascular disease, type 2 diabetes and osteoporosis. The prevalence of periodontitis ranges from 45.9% to 75.7%, as reported in some developed countries [2,3,4]. Meanwhile, osteoporosis affected 75 million unique individuals, including a third of the post-menopausal women in the United States, Europe and Japan [5]. Furthermore, both diseases share similar risk factors such as age, genetics, hormonal changes, calcium and vitamin D deficiency, and smoking, further suggesting that the bone loss in both diseases is most likely due to disruption of bone homeostasis. Owing to this potential mechanism, the interdisciplinary care of treating both diseases simultaneously may have a positive synergistic effect for favorable clinical outcomes [6]. For periodontitis, the initial treatment method includes mechanical debridement and antibiotic application. However, in most cases, it does not allow the regeneration of lost periodontal tissues. In addition, incomplete regeneration might result in residual pockets which can lead to worsening and relapse of periodontitis. Several methods have been introduced to treat residual pockets, such as adjunctive localized antimicrobials [6] and wide excision surgeries [7]. However, these methods have limited results; thus, scientists are exploring alternative methods for periodontal regeneration, such as tissue engineering. In fact, osteoporosis is an international public health problem as cases of osteoporosis are increasing gradually due to an increase in life expectancy [8]. Currently, it is estimated that over 250 million people are living with osteoporosis, with the majority of the patients being over 60 years old. Osteoporosis is a systemic degenerative bone loss whilst periodontitis involves local inflammatory bone loss due to an infectious breach of gingival and alveolar tissues. In addition, osteoporosis and periodontitis are both prevalent diseases characterized by bone resorption.

An efficient periodontal regeneration could be broadly classified into the formation of an epithelial seal, secretion of new cementum, fabrication of functionally oriented connective tissue fibers, and restoration of alveolar bone heights. As far as we know, epithelial cells surrounding the bone defects migrate faster than other competing cells such as mesenchymal stem cells, thus resulting in a long epithelial junction that prevents the formation of a new attachment on the diseased tooth. Therefore, the exclusion of epithelial cells is crucial to allow the progenitor cells to access the root surface. To put it another way, cells surrounding the periodontal defect whom had no function in forming new attachment tissues were blocked from any further contact with root surfaces. A guided tissue regeneration (GTR) method works by forming a physical barrier membrane between the periosteal flap and the root surface and initial results have shown that such a membrane was able to direct gingival connective tissue and migrating epithelium away from the root and thus was able to allow the regeneration of the progenitor cells such as cementoblasts and osteoblasts [9]. Furthermore, the presence of a physical membrane was also reported to provide stability for blood clots during the early stages of healing so as to ensure sufficient space for migration of periodontal apparatus. Both absorbable and non-absorbable biomaterials were used in GTR studies and non-absorbable materials are currently more generally accepted, as there is no need for a second surgery to remove the materials after regeneration [10]. Among them, collagen is particularly suitable for GTR applications due to its chemotactic capability and its stimulation in fibroblast proliferation. Moreover, collagen has been reported to be an excellent barrier for migrating epithelial cells and a good platform for vascular tissue penetration. Even so, these biomaterials should still possess ample mechanical integrity during the first 8 weeks of regeneration so as to prevent implant failure. In addition, the degradation of resorbable membranes should not interfere with the healing process. Furthermore, the implanted scaffold should be able to withstand the biting forces and harsh micro-environment of the periodontium so as to allow promising regeneration. In spite of having many advantages, there is a need to explore alternative ways to modify collagen [11]. Because currently, it is still challenging for collagen to meet all the clinical requirements in regeneration.

Bioceramics refers to a group of unique ceramic materials with excellent physiological behaviors and is commonly used in hard tissue regeneration [12,13]. Many studies have confirmed that calcium silicate (CS) are biologically active and are able to induce the formation of a hydroxyapatite layer on the surface of scaffolds after immersion in simulated body fluid, which enhances bonding between scaffold and the surrounding bone tissues [14,15]. Furthermore, CS had been shown to regulate mRNA expression of periodontal ligament cells via localized ion release, thus further promoting dentin metabolism and increased secretion of cementum by cementoblast [16]. In addition, CS had been shown to up-regulate bone-related proteins, such as bone sialoprotein (BSP) and osteocalcin (OC) in animal models of periodontal defects, thus strongly indicating that CS could enhance bone regeneration [17]. Previously, we had successfully 3D printed strontium-doped CS scaffolds (SrCS: Sr for strontium and CS for calcium silicate) and showed that it had enhanced biological, mechanical and osteogenesis capabilities as compared to the traditional CS [14]. Strontium (Sr) is one of the most important trace elements in the human body and is closely related to bone formation and regeneration [18]. Sr is commonly used as a therapeutic drug for the treatment of osteoporosis and it had been shown to be effective in preventing osteoporotic-related fractures [19]. In addition to effectively inhibiting bone resorption, Sr ions can also promote bone tissue formations due to its ability in stimulating bone precursors cells to synthesize collagen via the calcium-sensing receptor (CaSR) on osteoblasts [20].

An important aspect to be considered when designing a scaffold for periodontal regeneration is that the scaffold should mimic the compositions of the extra-cellular matrix of periodontal tissues, thus allowing for efficient regeneration. Periodontium is made up of cementum, periodontal ligament, alveolar bone and gingiva; the design of the scaffold should then be customized to enhance both hard and soft tissue regeneration. In 2015, Rasperini et al. fabricated a personalized scaffold for periodontal regeneration by co-printing multiple biomaterials using polycaprolactone and hydroxyapatite via a laser sintering technique [21]. The study has showed that application of 3D printing technologies could be used to fabricate scaffolds with mimicry to native structures and for large periodontal bone defects [22]. However, the long-term results of the study have showed that the fabricated scaffold had limited bone regeneration, further suggesting the need to consider other scaffold design or modification alternatives [23]. In the past decade, the evolution of 3D printing and additive manufacturing, especially multi-extrusion 3D printing, have been explored in tissue engineering and have showed potential as a valuable strategy for the development of multi-layer scaffolds [24]. A recent study by Porta et al. fabricated a porous multi-layer polycaprolactone with a Sr-hydroxyapatite scaffold via a single step extrusion process and proved that a multi-layer scaffold had improved biological, mechanical and osteogenic capabilities as compared to neat one-layer scaffolds [25]. In vitro studies had showed that the presence of ceramics within the polymeric matrix enhanced secretion of bone mineralization-related proteins. Furthermore, the single step fabrication process allowed excellent integrity between the different layers of the scaffold [26]. These studies conveyed the knowledge that the development of more complex and patient-specific substitutes, such as combining various known biomaterials and bio-active ions, are required to better match the overall structure of the native periodontal tissues.

To mimic the native hierarchy periodontium, the 3D-printed SrCS scaffold is used as a base to provide structural support for the formation of bone tissue whilst the gingiva fibroblast-laden collagen is used for supporting soft tissue regeneration and promoting bone mineralization. This study evaluates its potential in future periodontal engineering and clinical applications by assessing the mechanical and regenerative capabilities of the scaffolds in an osteoporotic animal model. In addition, the levels of factors released from the gingiva fibroblasts were also assessed to evaluate for the regeneration capabilities of the scaffolds. At this point in time, it is important to note that both osteoporosis and periodontal regeneration share similar growth factors and each growth factor assessed in this study was reported to have significant correlation between bone and periodontal tissue [27]. This cell-laden 3D fabricated bi-layer scaffold was found to have significantly enhanced bone regeneration capabilities in both in vitro and in vivo studies. We hoped that such a novel bi-layer scaffold could be effectively used in future tissue engineering studies of osteoporosis and periodontitis; in this way we may broaden its application.

## 2. Materials and Methods

### 2.1. Fabrication of SrCS Scaffolds

SrCS were synthesized according to methods published previously [14]. Firstly, CS was fabricated by sintering analytically graded 70% calcium oxide (CaO, Sigma-Aldrich, St. Louis, MO, USA), 25% silicon dioxide (SiO2, Sigma-Aldrich, St. Louis, MO, USA) and 5% alumina oxide (Al2O3, Sigma-Aldrich, St. Louis, MO, USA). To fabricate SrCS, the conditions of CS powders were changed in 20% strontium oxide (SrO, Sigma-Aldrich, St. Louis, MO, USA) to replace CaO. The green materials of CS and SrCS were sintered at 1400 °C for 2 h and then cooled to room temperature. The mixture was then grounded with agate milling balls in 99.5% ethanol (Retsch PM-100, Retsch GmbH, Germany) for 6 h and dried in an oven for 12 h. Prior to printing of the scaffolds, the SrCS powder was mixed into pure ethanol at a concentration of 0.1 g/mL, and subsequently dripped into melted polycaprolactone (PCL, Mol. Wt. 43,000 to 50,000, PolySciences, Warrington, PA, USA) and stirred till the ethanol evaporated. The paste was then loaded into a BioScaffolder cartridge (BioScaffolder 3.1, GeSiM, Großerkmannsdorf, Germany) and extruded via a 400 µm needle with 200–300 kPa and 1.5–2 mm/s.

### 2.2. Characterization of Physicochemical Properties of SrCS Scaffolds

X-ray diffractometry (XRD; Bruker D8 SSS, Karlsruhe, Germany) was applied to characterize for the composition of SrCS. The diffraction pattern was obtained on 2θ, ranging from 20 to 50 degrees with scanning steps of 1 degree/min. In addition, the microstructure of the scaffold was observed using a scanning electron microscopy (FE-SEM; JEOL JSM-6700F, Tokyo, Japan) with a lower secondary electron image setting and 3 kV acceleration voltage. Prior to the SEM tests, the scaffolds were covered with a nano-layer of gold.

### 2.3. Assessment of In Vitro Bioactivity

The scaffolds were placed into tubes containing DMEM to assess for levels of hydroxyapatite formation in order to assess for bioactivity of the SrCS scaffolds. The tubes were placed into 37 °C incubators for various durations. The scaffolds were then removed from the tubes, rinsed with de-ionized water and pure alcohol and dried in an oven. The hydroxyapatite formation was then observed using SEM with methods as described above. In addition, ions released into the medium, such as Ca, Si, P and and Sr ions, were measured using an inductive coupled plasma-atomic emission spectrometer (ICP-AES; Perkin-Elmer OPT 1MA 3000DV, Shelton, CT, USA).

### 2.4. Fabrication of Cell-Laden Col Hydrogel and Bi-Layer Scaffold

For the Col hydrogel, 3% (*w*/*v*) porcine (Sigma-Aldrich, St. Louis, MO, USA) was fully dissolved in phosphate buffered solution (Invitrogen, Grand Island, NY, USA) at 40 °C. Human gingiva fibroblasts (hGF, ScienCell Research Laboratories, Carlsbad, CA, USA) were used for subsequent in vitro studies. The cells were first cultured to passages 3 to 8 using the commercial medium and antibiotics provided by the manufacturer. Cells were trypsinized and collected with methods published previously and 5 × 10^5^ cells/mL were loaded into the Col hydrogel. The cell-laden Col hydrogel was then loaded with a BioScaffolder cartridge (BioScaffolder 3.1, GeSiM, Großerkmannsdorf, Germany) and extruded via a 400 µm needle with 10–20 kPa and 1.5–2 mm/s. The extruded hydrogel was then cross-linked using physical methods by lowering the printing temperature. The bi-layer scaffolds were fabricated using Bioscaffolder with SrCS scaffolds as the base and cell-laden Col hydrogel at the top in order to facilitate guided tissue regeneration.

### 2.5. Cellular Viability and Proliferation

A live/dead assay was used to quantify the cell viability and proliferation of hGF in Col bio-ink in one-lay or bi-layer scaffolds. The one-lay or bi-layer scaffolds were removed from the medium and rinsed gently with phosphate buffer solution after being cultured for 1, 2, 4, 6, and 8 weeks. Live/dead viability/cytotoxicity kits (Invitrogen, Grand Island, NY, USA) were used to stain the cells with fluorescence and a confocal microscope (Leica TCS SP8, Wetzlar Germany) was used to view the fluorescent cells. The membrane of live cells would be marked with green fluorescence, whilst the membrane of dead cells would be marked with red fluorescence. In addition, an MTT assay was used to determine for cellular proliferation. In brief, the cultured medium was removed and replaced with 5 mg/mL of MTT solution. The culture was then incubated for 3 h in the dark, followed by the addition of dimethyl sulphoxide for the dissolution of formed MTT crystals. A micro-plate reader was then used to analyze for absorbances of MTT. Three individual tests were conducted for the above test and the averages were recorded.

### 2.6. Secretion Protein Analysis

An enzyme-linked immunosorbent assay (ELISA) was used to analyze the levels of fibroblast growth factor-2 (FGF-2, ab246531, Abcam, Cambridge, MA, USA), bone morphogenetic protein 2 (BMP-2, EHBMP2, Invitrogen, Grand Island, NY, USA) and vascular endothelial growth factor (VEGF, ab222510, Abcam, Cambridge, MA, USA) in the medium of the scaffolds after a specified duration of culture. The ELISA kit (Invitrogen, Grand Island, NY, USA) was used according to the manufacturer’s instructions and was further correlated to a standard curve. Three individual tests were conducted for each sample. All the cultivation runs were performed three times in the experiments.

### 2.7. Osteogenesis Capabilities

In order to evaluate that hGF-laden Col bio-ink can promote osteogenesis, Wharton’s jelly mesenchymal stem cells (WJMSCs) were used here for the experiment. The cells were cultured on the SrCS side with commercial medium for 1 day and changed to osteogenic cell differentiation medium (StemPro™ osteogenesis differentiation kit, Invitrogen, Grand Island, NY, USA). The levels of Alkaline Phosphatase were used to analyze for the osteogenic capabilities of our scaffolds after being cultured for 3, 7, and 14 days. After specified durations of culture, 0.2% NP-40 was used for cell lysis, followed by 1500× *g* centrifugation for 10 min and an addition of para-nitrophenylphosphate (pNPP, Sigma-Aldrich, St. Louis, MO, USA) and 1 M diethanolamine buffer for substrate reaction for 15 min. Next, 5 N NaOH was added to quench the reaction and absorbance was subsequently measured at 405 mm using a plate reader. In addition, according to the manufacturer’s instructions, bone sialoprotein (BSP) and osteocalcin (OC) enzyme-linked immunosorbent assay kits (Invitrogen, Grand Island, NY, USA) were quantified the BSP and OC expression of WJMSCs cultured on SrCS and bi-layer scaffolds for 7 days and 14 days, respectively. All the cultivation runs were performed three times in the experiments.

### 2.8. Establishment of Osteoporotic Animal Models

The animals used in this study were New Zealand female white rabbits with an average weight of 2.0 kg and were fed and housed according to the protocol established by the Animal Experiment Center of China Medical University (CMUIACUC-2019-099-1). After a week of adaptation to the environment, the animals were anesthetized according to protocols and ovariectomy was conducted to induce post-menopausal-like symptoms. After the surgery, antibiotics were provided until the incision wound had healed. Next, intramuscular methylprednisolone sodium succinate at 1 mg/kg/day was given for 4 weeks to induce osteoporosis. The animals were then anesthetized and a skin incision was made along the lateral patella to expose the lateral femoral condyle. A cylindrical hole of about 7 mm in diameter and 8 mm in depth was drilled using a dental drill and the various scaffolds were then implanted into the gap. After the surgery, the animals were properly cared for as per post-operative protocols. The animals were then sacrificed and accordingly followed by subsequent analysis.

### 2.9. µCT Evaluation

The scaffolds were excavated with the surrounding encapsulated bone and scanned using a multi-scale X-ray nano-CT machine (SkyScan 2211, Bruker Belgium) with the following settings: 100 kVp, 30 µA and output of 20 W. A total of 2000 images were taken with slice increment of 16 µM. The images were then reconstructed using a software from Insta Recon (Bruker Belgium). The bone mass (BV/TV) and bone trabecular thickness (Tb.Th) were then calculated using AvizoFire 8.0 (Visualization Sciences Group, Bordeaux, France).

### 2.10. Histology Evaluation

Similarly, the scaffolds were excavated and sent for histological evaluation. Firstly, the scaffolds were fixed in 10% formalin for 2 days, rinsed with phosphate-buffered solution, embedded in paraffin and sliced into 6 µM slices. The sliced sections were then stained with Von Kossa (VK, Scytek Lab, USA) and hematoxylin and eosin (HE, Scytek Lab, USA) stains to identify new bone and calcified bone growth, respectively. A fluorescence microscope (Olympus BX51, Tokyo, Japan) was used to examine the stained tissues.

### 2.11. Statistical Analyses

A minimum of three independent tests was performed for each experiment, and all data in this study were reported as the mean ± standard deviation (SD). The Student’s *t*-test was used to analyze the significant differences between groups. In all experiments, the results were considered statistically significant if the *p*-value < 0.05.

## 3. Results and Discussion

### 3.1. The Characterizations of SrCS Scaffolds

**Figure 1:**
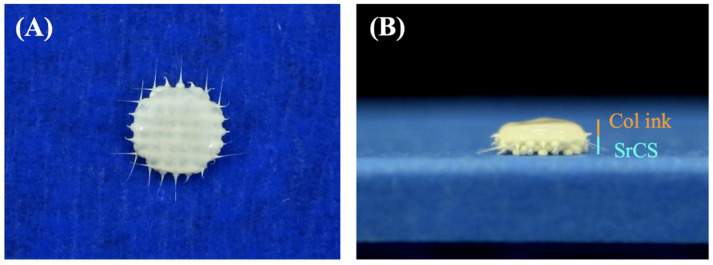
(**A**) Top and (**B**) side view photo images of the Col bio-ink/SrCS bi-layer scaffolds.

Calcium silicate-related materials are widely used in the treatment of periodontal diseases due to its excellent stability and osteoinductive capabilities. Therefore, scientists have attempted to modify CS in order to further enhance its osteogenic capabilities. Previously, we had successfully developed 3D-printed SrCS scaffolds and showed that such a modification significantly enhanced mechanical and osteogenesis as compared to traditional CS scaffolds [14]. However, it is insufficient to just regenerate the hard tissues component of periodontium while neglecting the soft tissues such as periodontal ligament and gingiva. By restoring the proper structure, the regenerated periodontium can then support tooth and bear biting forces and stress, thus mimicking the function of their natural counterparts. In this study, we attempted to fabricate a multi-layered hard-soft tissue scaffold composed of SrCS as the supporting base and Col bio-ink as the platform for soft tissue. The recent development of 3D printing technologies has allowed us to fabricate customizable scaffolds for various clinical applications. Photo images of the scaffolds were taken and shown in Figure 1. As can be seen, each strut and pore of the SrCS scaffold was uniform and the shearing tears of both the SrCS and the Col bio-ink were neat at each end. There has been an increasing number of reports and evidence stating that the porous scaffold had enhanced proliferation and differentiation as compared to scaffolds with no pores. It has been known since the early 2000s that scaffold geometries are critical in influencing cellular responses and bone regeneration. In addition, current studies have shown that pores up to few hundred μm are critical in enduring successful bone regeneration [28].

**Figure 2:**
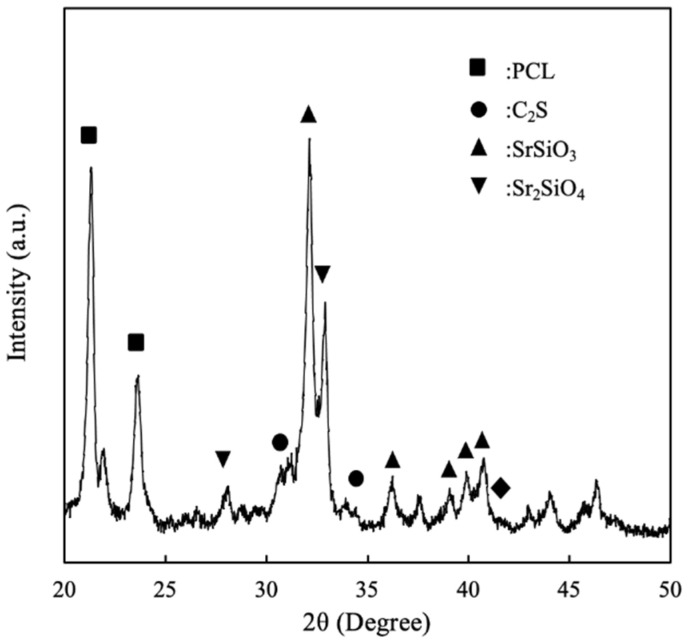
X-ray diffractometry (XRD) of SrCS scaffold.

The X-ray diffractometry analysis for SrCS was shown in Figure 2. As can be seen, there was presence of C2S, SrSiO_3_ and Sr_2_SiO_4_ peaks in the analysis [29], thus showing the successful incorporation of Sr ions into CS. Sr and calcium ions are part of the alkali metal group; thus both ions have very similar characteristics, especially in the area of bone metabolism [30]. Numerous studies have demonstrated that Sr ions have dual behaviors in simultaneously stimulating osteogenesis and inhibiting osteoclasts. Similarly to calcium ions, Sr ions can bind to calcium-sensing receptors found on osteoblasts, which would activate downstream mitogen-activated protein kinase (MAPK) pathways to enhance bone formation. On the other hand, Sr ions were found to inhibit the receptor activator of nuclear factor kappa-b ligand (RANKL), receptor activator of nuclear factor kappa-b (RANK) and osteoprotogerin (OPG) pathways, which further inhibit bone resorption by osteoclasts [18]. RANK and RANKL are involved in the differentiation of monoblasts and macrophages into the osteoclasts; thus, inhibition of such factors is critical in ensuring bone growth from the osteoblasts. Therefore, it is important that the scaffold must be able to release Sr ions in a sustained and controlled manner in order to achieve therapeutic effects [19]. Controlled release is critical to prevent cellular toxicity and thus subsequent tests were required to confirm for therapeutic effects of our scaffolds.

### 3.2. In Vitro Bioactivity

**Figure 3:**
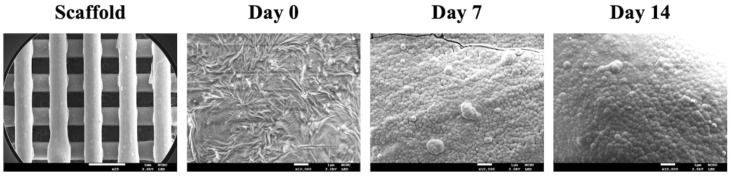
Field emission–scanning electron microscopy (FE-SEM) photographs of the SrCS scaffold before and after soaking in DMEM for different time-points. The scale bars of low-magnification and high-magnification SEM are 1 mm and 1 µm, respectively.

The microstructure of the surfaces of the scaffolds after immersion for various curtains were taken using SEM and shown in Figure 3. Conventional bioactive ceramics in the market were known to spontaneously form a layer of biologically active hydroxyapatite on the surfaces after transplanting into bone defects [31]. This layer is important as it is involved in integrating between the scaffold and bone tissues, thus numerous studies have attempted to coat hydroxyapatite onto surfaces of materials in order to improve osteoconductivity [32]. Recently, it was reported the Si-OH bonds on the surfaces provided negative charges that interact with the calcium ions in the simulated body fluid, thus resulting in the formation of amorphous calcium silicate [33], after which, the amorphous compounds then react with the phosphate ions to form hydroxyapatite. From the SEM images, it could be seen that surfaces of the SrCS were rough and each strut and pore of the scaffold were uniform (about 500 µm). It is common knowledge that rough surfaces improved short- and long-term behaviors of cells such as adhesion, proliferation and differentiation. After 7 days of immersion, it could be seen that spherical aggregates of hydroxyapatite were distributed sparsely over the entire surface of the scaffold. However, after 14 days of immersion, the aggregates were larger in size and covered a larger area of the scaffold compared to the 7 days of culture. The rough surfaces and the hydroxyapatite formation capability of the scaffold strongly indicated that SrCS could support proliferation and differentiation of osteogenic-related cells [34]. In addition, various reports made by others suggested that the level of hydroxyapatite formation could be used as a rough estimate of subsequent cellular activities.

**Figure 4:**
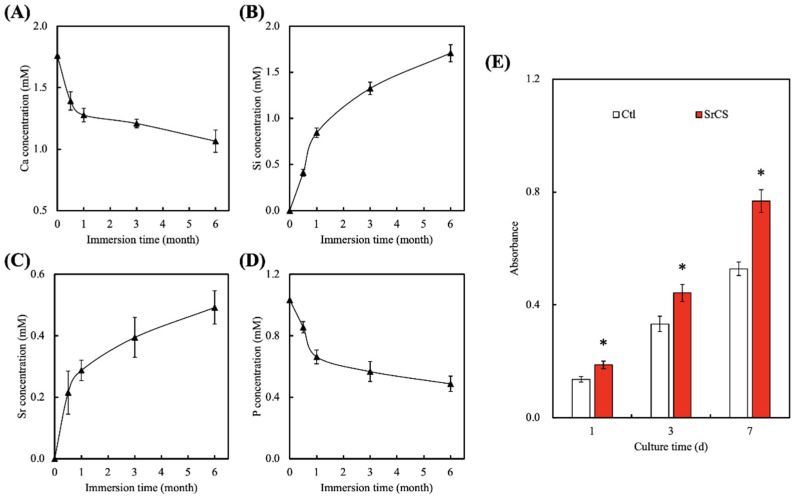
(**A**) Ca, (**B**) Si, (**C**) Sr, and (**D**) P ion concentrations in DMEM after immersion for different durations. (**E**) The proliferation of encapsulated hGF cultured under one-layer or bi-layer scaffolds for various time-points. * indicates significant difference (*p* < 0.05) from Ctl group. Data presented as mean ± SEM, *n* = 6 for each group.

An important aspect of a good scaffold is its ability to have sustained and controlled the release of ions into its surrounding. The ions released from the scaffolds were known to influence certain behaviors of cells and the profiles of several released ions were as shown in Figure 4. As can be seen, there was a sharp decline in the concentrations of Ca and P ions for the first 24 h of immersion, followed by a sustained decline up to 6 months of immersion [35]. On the other hand, there was a burst release in Si ions of about 0.5 mM and Sr ions of about 0.2 mM for the first 24 h of immersion, followed by a gradual release of ions up until 6 months of immersion. As reported by others, <2 mM of Si ions in the surrounding fluid was known to stimulate collagen production and enhance cellular proliferation [36]. It could be seen that after 6 months of immersion, the total accumulated amount of Si ions in the DMEM was 1.6 mM. In addition, Sr ions were reported to enhance osteogenic capabilities of cells via increasing secretion of ALP and OC [37]. Furthermore, an adequate presence of Sr ions was known to reduce and regulate inflammatory responses. Therefore, it is important that the scaffold is able to release various ions in a sustained manner; yet, at the same time, it must be controlled to avoid detrimental and toxic effects. Gao et al. reported that the release of alkaline ions into the environment enhances tissue–scaffold interaction due to the presence of amide bonds [38]. In addition, SrCS was able to significantly enhance the proliferation of WJMSCs as compared to the control (Ctl), which had approximately 1.3-, 1.3-, and 1.5-times the level of proliferation as compared to Ctl after 1, 3 and, 7 days of culture, respectively (Figure 4E). Kendler indicated the cell cultured medium with different ions regulated the osteoblast-like cells in increased extracellular matrix protein synthesis and promoted the proliferation and differentiation behavior of osteoblasts [39].

### 3.3. Cytotoxicity and Proliferation of hGF Laden in Col Ink

**Figure 5:**
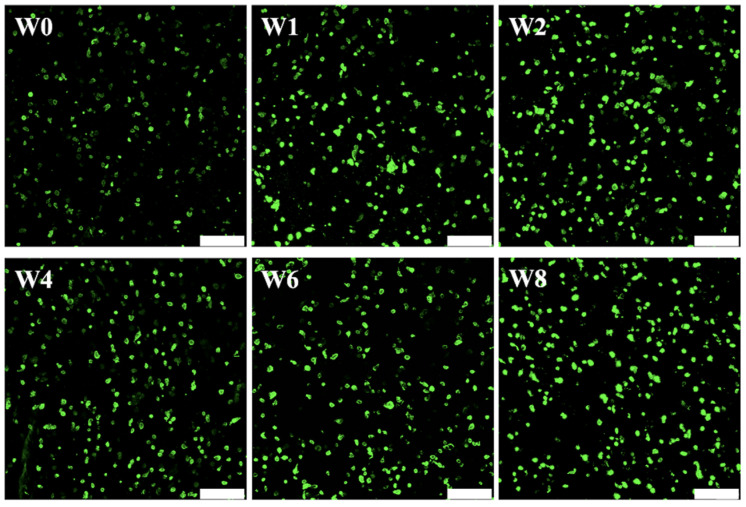
The fluorescent micrographs of the hGF-laden Col bio-ink before and after being cultured for different time-points. The scale bar is 200 µm.

The cytotoxicity test and cellular proliferation capability is an important factor to consider when choosing the bio-ink. Live/dead assay was conducted at weeks 0, 1, 2, 4, 6, and 8 and the results were shown in Figure 5. As can be seen, there were no dead cells (red fluorescence) at all time points, thus indicating that this Col-based bio-ink was non-toxic to hGF. Furthermore, hGF quantities doubled after 1 and 2 weeks of culture and increased steadily over the rest of the culture duration. In addition, the cells were homogeneously encapsulated in Col-based bio-ink, which is important to allow efficient regeneration. The success of Col-based bio-ink depends on the composition and microstructure of the hydrogel. Thus, it would be ideal if we are able to mimic the native structure of the tissue. Collagen is the main component in most soft tissues, including periodontal ligament and gingiva [40]. In addition, collagen forms part of the extracellular matrix and thus the interactions between collagen and cells are vital in regulating cellular behaviors. Collagen has been examined as the ideal matrix to develop bi-layer scaffolds due to its outstanding biocompatibility and capacity to improve cell proliferation and differentiation, inducing several signal pathways to prove specific tissue-related protein expression [41]. Furthermore, as a natural molecule, collagen is biodegradable, biocompatible, easily available, and highly versatile, therefore making it an excellent polymer for tissue engineering [42].

**Figure 6:**
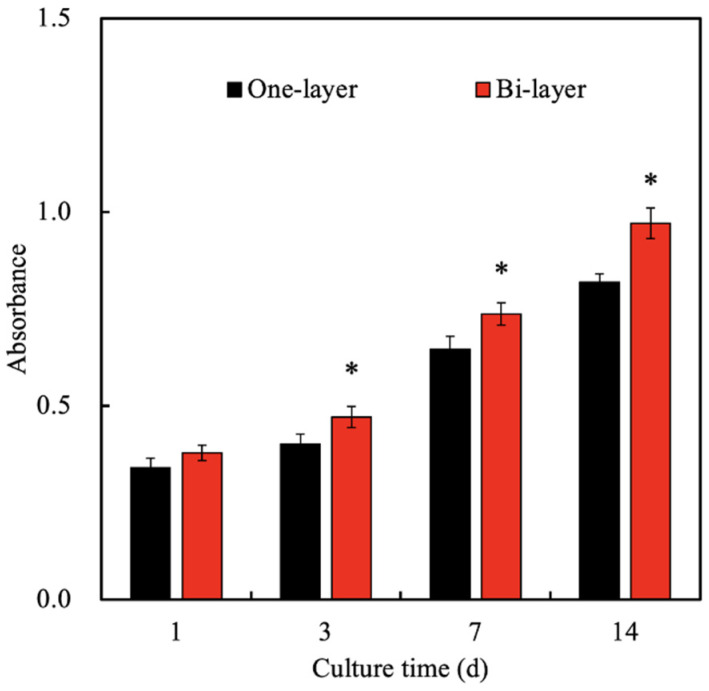
The proliferation of encapsulated hGF cultured under one-layer or bi-layer scaffolds for various time-points. * indicates significant difference (*p* < 0.05) from the one-layer group.

Previously, we had successfully fabricated the SrCS-contained gelatin methacrylate hybrid scaffold using 3D printing via a UV light curing processing [37]. From that study, it could be noted that the incorporated SrCS released Si and Sr ions that enhanced cellular proliferation and differentiation. Therefore, in this study, we fabricated a novel bi-layer scaffold comprising of SrCS as the base with a Col bio-ink covering the top of the scaffold. Similarly, we had previously fabricated SrCS scaffolds and results had shown that SrCS had the potential to stimulate both osteogenesis and angiogenesis. The concept behind this novel Col/SrCS bi-layer scaffold was to imitate the native structures of periodontium, which is also a bi-layered tissue comprised of soft and hard tissues. Currently available biomaterials in the market are mainly focused on regeneration of the hard tissues of periodontium such as the cementum. However, the soft tissues covering the cementum are equally important in maintaining the integrity and structure of the tooth. Therefore, the cellular proliferation level of hGF-laden Col/SrCS scaffolds was quantified and the results were as shown in Figure 6. As seen, proliferation levels were slightly higher in the bi-layer group as compared to the one-layer Col bio-ink. However, there were no significant differences in the level of proliferation at day 1 of culture. On the other hand, significant differences were noted at all the time points of day 3, 7, and 14 days of culture. Proliferation levels were approximately 1.2- to 1.4-times higher in the bi-layer groups after 3 days of culture. Even though Col bio-ink is a good substrate for cell encapsulation, it could be seen that co-fabrication of SrCS further enhanced cellular proliferation, thus indicating that there is a further need for evaluation of other combinations. It was hypothesized that the release of Si and Sr ions from SrCS further promoted the proliferation behavior of hGF [43]. In a previous study, Yamaguchi-Ueda indicated that a combination of ions, which includes Sr and Si ions were able to stimulate gingival cell migration via the ERK1/2 pathway [44]. These results clearly showed that dental materials containing multiple ions or mechanisms is useful for future tissue regeneration.

### 3.4. Quantification of FGF-2, BMP-2, and VEGF Secreted from hGF Encapsulated in One-Layer or Bi-Layer Scaffold

**Figure 7:**
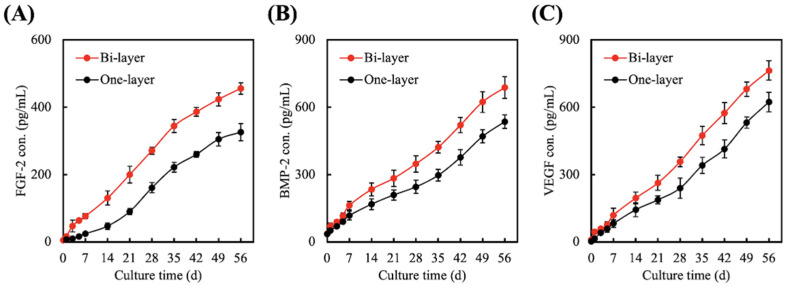
Evaluation of (**A**) FGF-2, (**B**) BMP-2, and (**C**) VEGF secreted from hGF encapsulated in one-layer or bi-layer scaffolds for different time-points.

In this study, the growth factors such as FGF-2, BMP-2, and VEGF were quantified using ELISA and the results were as shown in Figure 7. It could be noted that the secretion profiles were similar in both groups; however, the bi-layer group had higher secretions at all time points up to 56 days of culture. It is important to note that our bi-layer scaffold had the potential to induce a significantly higher amount of FGF-2 (Figure 7A), BMP-2 (Figure 7B), and VEGF (Figure 7C), even after 56 days of culture. Depending on the extent of periodontal tissue injuries, healing and regeneration of tissues were reported to take up to 4 to 8 weeks. FGF-2 is known to be involved in the regeneration of new alveolar bone, cementum, and even periodontal ligaments. In an animal model with periodontal defects, it could be seen that the application of topical 0.3% FGF-2 increases regenerated tissue and cells after 3 days and angiogenesis could be noted after 7 days. Furthermore, it was concluded that FGF-2 enhanced expression of BMP-2 and osteogenic-related markers such as osterix, OC and ALP, thus enhancing regeneration of periodontal tissues [45]. On the other hand, BMP-2 is another compound commonly used in periodontal regeneration. Studies have attempted to use a BMP-2 gene delivery system and even coated biomaterials with recombinant human BMP-2 in order to enhance periodontal regeneration. All studies have shown that BMP-2 were involved in periodontal regeneration and that the application of BMP-2 into an animal model of periodontal defects demonstrated a significant regeneration of hard tissues. BMP-2 were reported to enhance the differentiation of chondroblasts and osteoblasts, thus resulting in new bone and cementum formation via Smad 1/5/8 pathway [46]. The activation of intracellular Smad-related proteins is known to affect cell differentiation-related genes and protein expression that was sensitive to the cultured microenvironment [47]. In addition, the formation of a vascular network is critical to ensure oxygen supply and nutrient supplementation. Most engineered scaffolds thus far were non-vascularized, thus forcing the transplanted scaffold to depend on the diffusion of oxygen from the recipient tissue. In such cases, the size of the scaffold had to be limited to ensure sufficient oxygenation and nutrition to avoid transplant failure. VEGF is the factor dominantly involved in angiogenesis, as it is a potent inducer of endothelial cell migration and proliferation [48]. With the results above, it can be seen that our novel bi-layer scaffold has the potential to induce osteogenesis, cementogenesis, and angiogenesis.

### 3.5. Effect of hGF-Laden Col Bio-Ink on Osteogenesis

**Figure 8:**
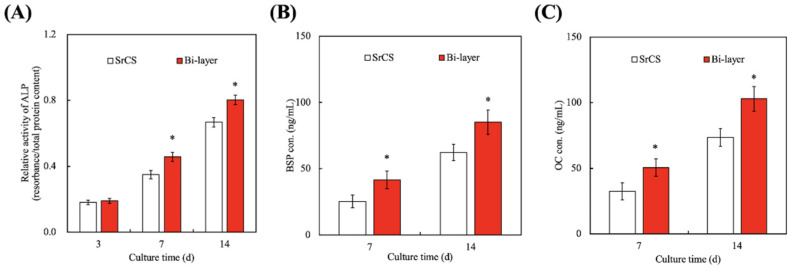
(**A**) The ALP, (**B**) BSP, and (**C**) OC proteins expression of WJMSCs cultured on SrCS and bi-layer scaffolds for different time-points. Data presented as mean ± SEM, *n* = 6 for each group. * indicates a significant difference (*p* < 0.05) when compared to SrCS.

Cells would secrete extracellular proteins that would self-assemble and develop into a suitable structure for cells to stay and mature in [49]. Cells were known to secrete different types of factors that influence certain downstream behaviors and activities. In the case of bone regeneration, ALP, BSP, and OC were common factors used in assessing levels of bone regeneration, as studies have proven that the abovementioned factors were involved in bone regeneration. As seen in Figure 8A, there were no significant differences in the levels of ALP secretion between the SrCS scaffold and the bi-layer scaffold at day 3. However, there were significant differences in secretion of ALP, BSP (Figure 8B), and OC (Figure 8C) from the bi-layer scaffold at 7 and 14 days. ALP is often detected in the early stages of bone retraction and thus is used to measure levels of early osteogenesis. On the other hand, BSP is found in mineralized tissues such as dentin, but the exact mechanism and involvement of BSP in bone regeneration is still being investigated [50]. OC is secreted by osteogenic-related cells during osteogenesis and is known to act on calcium ions to bring about mineralization. Furthermore, OC is also commonly used as a bone density marker to evaluate for bone regeneration and tissue formation [51]. According to these protein expressions related to bone regeneration, it is confirmed that WJMSCs cultured in the bi-layer scaffold can have better bone differentiation behaviors. And based on the previous results, it is speculated that the growth factors (FGF-2, BMP-2, and VEGF) released by hGF in Col ink can effectively enhance the osteogenesis of WJMSCs.

### 3.6. In Vivo Bone Regeneration

**Figure 9:**
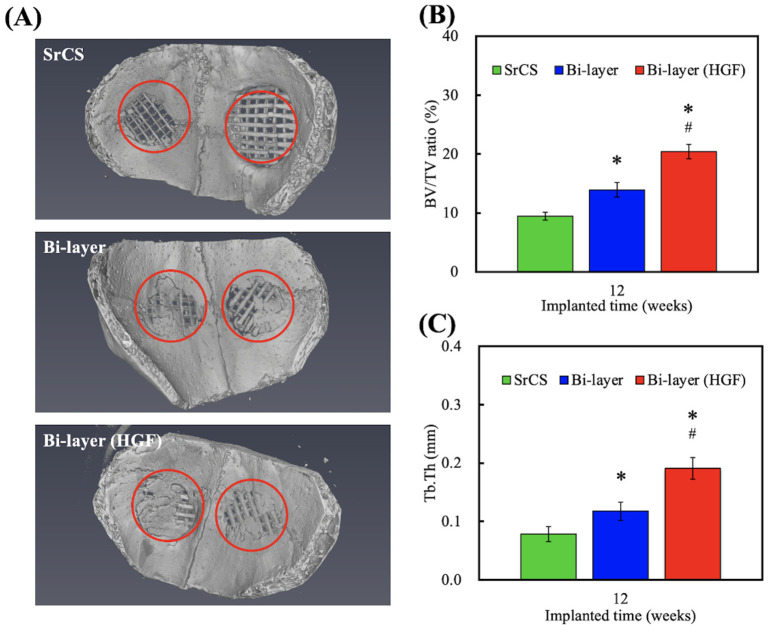
(**A**) Representative µCT images of osteoporotic rabbits’ cranial bone defect model with different 3D-printed scaffolds after implanted for 12-weeks. The µCT-quantified histograms of (**B**) bone volume/total volume (BV/TV) and (**C**) trabecular thickness (Tb.Th). * indicates a significant difference (*p* < 0.05) when compared to SrCS. # indicates a significant difference (*p* < 0.05) when compared to bi-layer.

The in vivo regenerative capabilities of the bi-layer scaffolds for osteoporosis periodontal defects were further investigated using animal models. After 12 weeks of implantation, the tissue surrounding the scaffold was excised and assayed by µCT imaging and computer-aided quantification of TB.Th and BV/TV ratios, as shown in Figure 9. As seen in the results, there were few opacities amongst the struts for SrCS0 as compared to the hGF-laden bi-layer scaffold, strongly suggesting that the bone tissue had not yet begun to regenerate (Figure 9A). From the µCT images, it could be seen that there were new bones in the core of the bi-layer with the hGF scaffold and that most of the struts of the scaffold were interrupted and invaded by ingrowth of new bones. On the other hand, the SrCS scaffold only had new bone growth at the periphery of the scaffold. The results showed that even after 12 weeks of implantation, the structure of the scaffolds was still maintained due to the slow degradation of SrCS. These µCT images clearly showed that our porous scaffold could guide tissue regeneration and that the material degrades upon the generation of new tissues. The above results may be because growth factors secreted from hGF accompanied by Sr ions, which leads to a synergistic effect on the promotion of new bone formation in vivo. Previous studies have also reported the effects of growth factors secreted from cells on the biological functions of osteogenesis [52]. The size of our pores was determined according to our previous studies and by reports made by others. Zheng et al. reported that macro-porous scaffolds of about 100 and 600 μm allowed better integration with the host bone tissue and also for subsequent vascularization and bone distribution [53]. Quantitative results were computed to further confirm the percentage of bone growth. As seen in Figure 9B, bone volume fractions were significantly higher in the hGF-laden bi-layer scaffold. Bone volume fraction indicates the area that is occupied by mineralized bone; therefore, the hGF-laden bi-layer scaffold had 20% of new mineralized bone whilst the nest bilayer scaffold had 13% and SrCS had 9%. This result strongly indicated that hGF-laden Col-ink was able to promote both in vitro and in vivo bone formation. In addition, trabecular thickness was also quantified to measure the 3D structure of cancellous bone, which could be used to predict the extent of bone regeneration (Figure 9C). Similarly, the hGF-laden bi-layer scaffold had significantly higher amounts of trabecular thickness than the other two groups.

**Figure 10:**
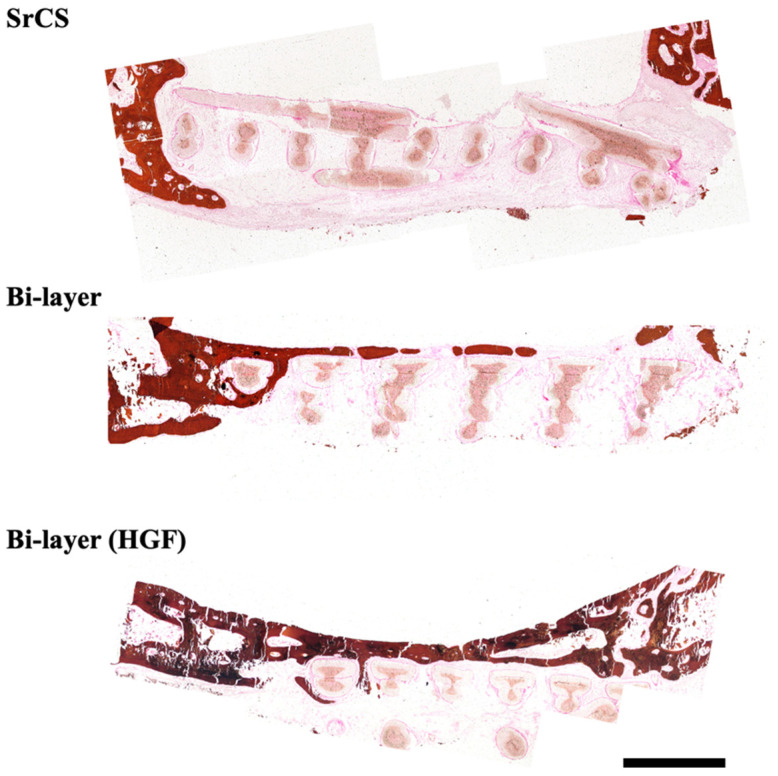
Histological analysis of new bone regeneration around and within the scaffolds in the osteoporotic rabbits’ cranial bone defect model. The Von Kossa (VK) stain of regenerated bone mass after 12 weeks of regeneration in the in vivo experiment. The scale bar is 1 mm.

The von Kossa staining (VK) of the harvested scaffolds after 12 weeks of implantation were shown in Figure 10. The histological sections confirmed the results from the µCT image. The SrCS scaffold only had new bone growth at the periphery of the scaffold, whilst the bi-layer scaffold with hGF were wrapped by new bone tissues and the integrity of the struts were invaded by bone tissues. Furthermore, the intensity of the VK staining for the hGF-laden bi-layer scaffold were stronger than the rest of the group, thus representing more bone growth. In a previous study, we fabricated the 3D-printed SrCS scaffold and demonstrated that the naked scaffold was not promoted as the bone regeneration in the osteoporosis rabbit tibia defect model [22]. In the SrCS scaffold, there was little evolution of bone deposition in the bone matrix, highlighting the importance of having a growth factors-contained scaffold for stimulation of osteo-progenitor cell differentiation and regeneration of osteoporosis tissue [54]. The interesting phenomenon to be noted is that the addition of hGF allowed for an increased bone tissue regeneration into the center of the scaffold. hGF is the most common cell source in the periodontal tissue and can be easily extracted for gingival tissue. The results demonstrated that the hGF-laden bi-layer scaffold could support critical bone defect regeneration and supply a good microenvironment in which to enhance the repair of damaged tissue. Their osteogenic capabilities had not yet be clearly understood, but previous studies have shown that BMP-2 and FGF-2 had a certain potential to influence hGF differentiation into osteoblasts [55,56]. Cho et al. treated hGF with 5-aza-2′-deoxycytidine and BMP-2 that successfully influenced hGF to differentiate to osteoblast lineage [57]. In addition to the traditional bioceramic materials-regulated bone regeneration, other research has indicated that growth factors, such as BMP-2 and VEGF treatment, enhanced bone formation in the defects within the first few weeks, thus indicating that growth factors may play the role in affecting bone regeneration and formation [58]. In our study, we hypothesized that the fabrication of a novel bi-layer scaffold could enhance expression of FGF-2 and BMP-2 from hGF, which in turn induces differentiation of hGF into osteoblasts for enhanced bone tissue regeneration [59]. The bone tissues observed in the middle of the bi-layer with the hGF scaffold could be attributed to the migration of osteoblasts via the pores, thus leading to subsequent enhanced bone tissue regeneration. In addition, the increased secretion of VEGF allowed efficient vascularization to reach the core of the pores, thus similarly leading to increased regeneration. To the best of our knowledge, this is the first time that the combination of hGF and cell-laden scaffolds have been assayed in an osteoporotic rat critical bone defect model. The molecular alterations of hGF are reflected on the production of osteogenic-related growth factors, leading to significant regeneration in self-repair and an enhanced differentiation. Therefore, future studies are needed to optimize the cell-laden condition and considered the regenerative capacity of the system and enhancing the mineralization of the newly developed bone tissue.

## 4. Conclusions

In this study, we fabricated a gingival fibroblast cell-laden collagen/strontium-doped calcium silicate (SrCS) bi-layered scaffold for periodontal regeneration. It could be seen from the XRD results that SrCS had retained the structural characteristics of CS after modification with Sr. In addition, the SrCS scaffold had hydroxyapatite formation after 7 days of immersion and the scaffolds were able to have a sustained release of Sr and Si ions up until 6 months of immersion. The ability to release ions over a period of 6 months was an important factor to support regeneration over a sustained period, as different tissues have different durations of regeneration. The collagen hydrogel was able to support proliferation and encapsulation of human gingival fibroblasts as seen from the immunofluorescence results. In vitro studies showed that the bi-layered scaffolds enhanced secretion of FGF-2, BMP-2, and VEGF from human gingival fibroblasts and ALP, BSP, and OC from WJMSCs. It could be hypothesized that the bi-layered scaffolds were able to enhance angiogenesis and osteogenesis due to upregulation of both angiogenic and osteogenic-related proteins and factors. From the in vivo studies, it could be noted that the bi-layered scaffolds had enhanced bone regeneration as compared to SrCS alone. The bi-layered scaffolds with human gingival fibroblasts had significantly higher Tb.Th and BV/TV ratios as compared to the bi-layered scaffolds and neat SrCS scaffolds after 12 weeks of implantation. From the computed tomography and histological findings, it could be noted that new bone tissues had started to grow and invaded the scaffolds itself. These results suggested that 3D-printed hGF-laden/SrCS scaffolds could enhance bone tissue engineering and show potential for use in future clinical applications. 

## Data Availability

Data are available in a publicly accessible repository.
